# Comparison of Organosulfur and Amino Acid Composition between Triploid Onion *Allium cornutum* Clementi ex Visiani, 1842, and Common Onion *Allium cepa* L., and Evidences for Antiproliferative Activity of Their Extracts

**DOI:** 10.3390/plants9010098

**Published:** 2020-01-13

**Authors:** Željana Fredotović, Barbara Soldo, Matilda Šprung, Zvonimir Marijanović, Igor Jerković, Jasna Puizina

**Affiliations:** 1 Department of Biology, Faculty of Science, University of Split, R. Boškovića 33, 21000 Split, Croatia; 2 Department of Chemistry, Faculty of Science, University of Split, R. Boškovića 33, 21000 Split, Croatia; barbara@pmfst.hr (B.S.); msprung@pmfst.hr (M.Š.); 3 Department of Food Technology and Biotechnology, Faculty of Chemistry and Technology, University of Split, R. Boškovića 35, 21000 Split, Croatia; zmarijanovic@ktf-split.hr; 4 Department of Organic Chemistry, Faculty of Chemistry and Technology, University of Split, R. Boškovića 35, 21000 Split, Croatia; igor@ktf-split.hr

**Keywords:** *Allium cornutum* Clementi ex Visiani, 1842, *Allium cepa* L., organosulfur compounds, amino acids, antiproliferative activity, apoptosis

## Abstract

Species that belong to the genus *Allium* have been widely used for human food and traditional medicine. Their beneficial health effects, as well as the specific aroma, are associated with their bioactive chemical compounds, such as sulfur compounds and flavonoids. Gas chromatography and mass spectrometry (GC–MS) and reverse-phase high-performance liquid chromatography (reverse-phase HPLC) were used to identify organosulfur and amino acid content of triploid hybrid onion, *Allium cornutum* Clement ex Visiani, 1842, and common onion, *Allium cepa* L. *Allium* extracts were tested for their antiproliferative activity in three human cancer cell lines (HeLa, HCT116, and U2OS). DNA fragmentation and DAPI staining analysis were performed on HeLa cells to evaluate the effect of extracts on DNA damage and cell morphology. The mRNA expression of p53, Bax, and Caspase-3 genes involved in apoptosis were analyzed by real-time PCR. Using GC–MS, 27 compounds were found in two *Allium* species headspaces. Differences were noted among the main compound abundance in the headspace (although the major thiols and disulfides were qualitatively identic in both *Allium* species) and dipropyl disulfide, diisopropyl trisulfide, and (Z)-prop-1-enyl propyl trisulfide were predominant sulfides. Identification of amino acids and their quantities were determined by reverse-phase HPLC. Most abundant amino acids in both onions were arginine (Arg) and glutamic acid (Glu). The results of cytotoxicity testing confirmed antiproliferative effects of both species. The DNA fragmentation assay, DAPI staining and real time PCR analysis confirmed that *A. cornutum* and *A. cepa* extracts induced apoptosis in HeLa cells. This study presents the evidence for possible therapeutic use of *A. cornutum* and *A. cepa* extracts against human cervical carcinoma cell line.

## 1. Introduction

The *Allium* genus includes more than 750 species of which about a dozen are economically important and cultivated as crops, garden vegetables, or ornamental plants. Annual production and consumption of onion, garlic, leek, chives, and their relatives, makes these *Allium* species among the most important vegetables in the world [1]. Except in diet, these plants have been used for many years in traditional medicine for the treatment and prevention of different diseases, like skin diseases, microbial infections, gastrointestinal disorders, worms, wounds, and tumors [2]. Nowadays, many epidemiological studies confirmed the connection between the consumption of *Allium* vegetables and decrease risk for development of many diseases, such as coronary heart disease [3,4], gastrointestinal diseases [5,6,7], different type of cancer [8,9], and inflammatory diseases [10,11]. These biological effects are associated with their chemical constituents, mainly sulfur-containing compounds and flavonoids. Flavonoids are responsible for the color of onion skin. They possess a strong antioxidant activity which makes them one of the best antioxidants and scavengers of free radicals [12,13]. After the consumption of onions, flavonoids are absorbed primarily from the stomach or/and small intestine [14]. Flavonoid glucosides are found to be significantly elevated in plasma after the ingestion of onion meal. Moreover, there was a strong correlation between the higher level of flavonoids in plasma and increased resistance of DNA to oxidative DNA damage, which is direct evidence of their antioxidant activity [15]. Volatile sulfur compounds exhibit even stronger antioxidant properties. They are responsible for extraordinary antimicrobial, anti-inflammatory, and anticancer effects. Different organosulfur compounds exhibiting biological activity have been reported in the common onion (*Allium cepa* L.) and garlic (*Allium sativum* L.). Diallyl disulfide, diallyl trisulfide, allyl methyl trisulfide, diallyl sulfide, and diallyl tetrasulfide are found mostly in garlic essential oil, while dipropyl disulfide, dipropyl trisulfide, methyl propyl trisulfide, dipropyl tetrasulfide, and prop-2-enyl propyl disulfide are found mostly in common onion [16,17]. Edible onion parts also contain carbohydrates, glucose, and fructose, while the skin is rich in galactose and arabinose. In addition, amino acids contribute to the sensory response and to the characteristic “umami” taste [18]. Not only might free amino acids be used in the protein formation, but they also happen to be an important nitrogen reservoir, which additionally contributes to the nutritional value of the *Allium* species [12]. Arginine (Arg) is an amino acid which appears to be present in huge amounts in onions, which correlates with the hypothesis that onions use Arg as a nitrogen reservoir [19]. Besides Arg, the amounts of glutamic acid (Glu) and lysine (Lys) are also prominent. Similarly, the main free amino acids present in garlic are glutamine (Gln), asparagine (Asn), Glu, and Lys [20]. In contrast, Lawson (1996) [21] found arginine as the most abundant amino acid present in garlic cloves. *Allium cornutum* is a minor garden onion crop traditionally cultivated in Asia, Europe, and North America, under different local names [22,23]. It is characterized by an unusual genetic structure and triploid karyotype (2n = 3x = 24), a result of a spontaneous hybridization between the three different *Allium* species [24,25]. Since the composition of headspace-volatile sulfur compounds and free amino-acids of triploid onion *A. cornutum* has not been studied so far, we aimed to identify those two groups of phytonutrients and compare them with the corresponding components of common onion. In addition, we confirmed the cytotoxic effect of extracts of both *Allium* species on three cancer cell lines: HeLa, HCT116, and U2OS. It is important to highlight that this was the first study of antiproliferative activity of hybrid onion, *A. cornutum*, and common onion, *A. cepa*, against these cancer cell lines. To better understand the molecular mechanism underlying the cancer cell’s growth arrest and death, we combined fluorescent microscopy, gel electrophoresis and gene expression analysis by real-time PCR in HeLa cells. Our results confirmed that both onion extracts induced an apoptotic process in treated cancer cell lines.

## 2. Results and Discussion

### 2.1. Headspace Volatiles of Allium Species

*Allium* species contain cysteine sulfoxides as precursors of volatile aroma compounds, stored in the cytoplasm of intact bulbs. After disruption of onion tissue, the alliinase enzyme cleaves cysteine sulfoxides into the sulfinic acids, which are immediately converted to thiosulfinates. Thiosulfinates are chemically unstable and undergo dissociation and rearrangement to the volatile sulfur compounds, responsible for specific onion odor [26,27]. Table 1 shows the volatile compounds identified from two freshly cut *Allium* species, *A. cornutum* and *A. cepa* L., obtained by headspace solid-phase microextraction (HS-SPME). A total of 27 compounds were identified in both species by gas chromatography and mass spectrometry (GC–MS) analysis. Differences were noted among the main compound abundance in triploid and common onion (although the major thiols and disulfides were qualitatively identic). Dipropyl disulfide, diisopropyl trisulfide, and (Z)-prop-1-enyl propyl trisulfide were predominant sulfides in *A. cornutum*, followed by a minor abundance of other sulfides such as (E)-prop-1-enyl propyl trisulfide, methyl methylthiomethyl disulfide or allyl propyl disulfide (Table 1). 

Another abundant compound in *A. cornutum* was 1-propanethiol accompanied with a minor abundance of prop-2-en thiol and methanethiol. In comparison with *A. cepa*, the abundance of several important compounds was higher: dipropyl disulfide (1.5 times), 1-propanethiol (1.1 times), and methyl methylthiomethyl disulfide (5.6 times). On the other hand, several compounds in *A. cornutum* were present with lower percentages (Table 1), such as (E)-prop-1-enyl propyl disulfide, (E)-prop-1-enyl propyl trisulfide, and trans-3,6-diethyl-1,2,4,5-tetrathiane. To our knowledge, this was the first analysis of volatile sulfur compounds in *A. cornutum*. Interestingly, dipropyl disulfide was previously reported to be the predominant compound in *A. cepa* L. [28,29,30], but it is the compound that highly dominates in *A. cornutum*, as well, with 31.9% (Table 1). Colina-Coca et al. (2013) [31] identified 12 volatile sulfur compounds in processed onion, using HS-GC–MS. The most dominant sulfur compound identified was dipropyl disulfide, with 48%, which is in correlation with the results of present study (Table 1, no. 18). Besides dipropyl disulfide (35.45%), which was the most dominate sulfur compound identified in fresh onion, Liu et al. (2018) [32] also reported a certain amount of 1-propanethiol, which was found to be abundant in *A. cornutum* (14.1%) and *A. cepa* L. in the current study. The second major compound present in *A. cornutum* (Table 1) was diisopropyl trisulfide, which was also reported to be the second most prominent compound present in *A. cepa* L., according to Ndoye Foe et al. (2016) [17] and Teshika et al. (2018) [33]. Two heterocyclic sulfur compounds, cis-3,6-Diethyl-1,2,4,5-tetrathiane and trans-3,6-diethyl-1,2,4,5-tetrathiane, contribute to specific flavor of raw and processed onion. It was noticed that enzyme alliinase can convert non-aroma compounds into the aroma compounds, at optimal temperature around 40 °C. The lower concentration of heterocyclic alkyl-substituted sulfur compounds (cis- and trans-3,5-diethyl-1,2,4-trithiolanes) in spring onion (*Allium fistulosum* L.) and chives (*Allium schoenoprasum* L.) in contrast to common onion (*A. cepa* L.) was due to thinner tissue diameters, which could inactivate alliinase almost immediately. These results suggest a thermal enzymatic formation pathway of heterocyclic sulfur compounds [34]. Similarly, the higher concentration of cis-3,6-diethyl-1,2,4,5-tetrathiane and trans-3,6-diethyl-1,2,4,5-tetrathiane in *A. cepa* L. in contrast to *A. cornutum* might be due to thinner tissue of triploid onion and possibly a different concentration of heterocyclic precursors. In the present study, this component was found to be minor in the headspace of *A. cornutum* (0.6%) and *A. cepa* L. (0.9%). In the headspace of *A. cepa* L., (E)-prop-1-enyl propyl disulfide was present with 1.7%, with typical flavor of cooked onion. In contrast to common onion, triploid onion headspace contained only 0.1% of this component. In general, there are four types of diallyl sulfides found in onions [35]: diallyl monosulfide (DMS), diallyl disulfide (DDS), diallyl trisulfide (DTS), and diallyl tetrasulfide (DTTS). The composition and amount of volatile sulfur compounds, known as “secondary aroma compounds”, can be influenced by the extraction conditions [28]. It has also been shown that the number of cysteine sulfoxides, the precursors of volatile sulfur compounds, increased by hybridization of common onion with some wild *Allium* species [36,37]. Despite the differences in the volatile sulfur composition, many components are highly similar for both species, as expected, since they are genetically closely related [38]. The volatile sulfur compounds identified in current study are known to be responsible for typical flavor and odor of onions [27,39], as well as for their strong biological activity [17,40,41,42,43,44]. Some of the relevant *Allium* species with their volatile sulfur compounds are described in the following section (Data S2).

### 2.2. Amino Acid Composition of Onion Bulbs

A total of 17 amino acids were found in two *Allium* species. Table 2 shows the list of all amino acids in onions and their content in mg/g of dry weight (DW). This is the first analysis of amino acid composition in triploid onion, *A. cornutum*. The most abundant amino acids in both species were Arg with 16.492 and 12.213 mg/g DW of total free amino acids in *A. cornutum* and *A. cepa*, and Glu with 14.964 mg/g DW of total amino acids in *A. cornutum* and 11.238 mg/g DW in A. cepa. These results are consistent with those of Lee et al. (2009) [45], where Arg was reported as the most abundant amino acid in different onion cultivars.

The amino acid analysis of *A. caepa* L. var Tropeana (red onion) seeds showed large amounts of Glu and Arg and lower amounts of Tyr and Asp [46]. Hansen (2001) [47] concluded that content of Arg and Gln comprised about 60% of total free amino acids in onions with much lower amount of Glu, which we found to be the second most prominent amino acid in triploid and common onion. The analysis of the amino acid content in *A. tuberosum* seeds revealed Glu and Arg as the two most prominent amino acids. They also found a relatively high amount of aspartic acid (Asp) [48], which is in correlation with our results. These two dominating amino acids, especially Arg, seems to have a crucial role as a source of nitrogen, which is an extremely important amino acid during the period of maturation [19]. Moreover, Glu and Arg might be responsible for the color of onion seeds [46]. It has been shown that Glu, the second most abundant amino acid found in both species in the present study, is the most important “umami” substrate responsible for specific onion taste [18,49].

### 2.3. Antiproliferative Activity of A. cornutum and A. cepa Extracts

**Figure 1:**
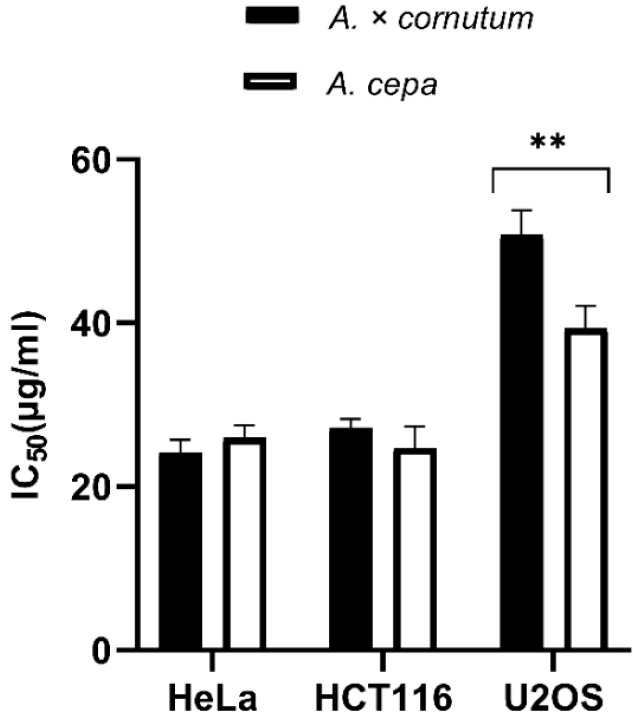
Antiproliferative activity of *A. cornutum* and *A. cepa* extracts against three human cancer cell lines: Hela, HCT116, and U2OS. The statistical significance was assessed by two-way ANOVA, followed by Dunnett’s multiple-comparisons test. The presented IC_50_ are mean values of at least three independent experiments performed in quadruplets, with SD presented with error bars, and significant levels comparing *A. cornutum* with *A. cepa* are marked as ** *p* < 0.01 (Data S4).

The results of MTS cell proliferation assay performed with *A. cornutum* and *A. cepa* extracts for the first time on HeLa, HCT116, and U2OS cancer cell lines are shown in Figure 1. The cells were treated with onion extracts for 48 h, and the results were expressed as IC_50_ values (50% cell growth inhibitory concentrations). HeLa and HCT116 cell lines showed high susceptibility to both extracts. HeLa cells were equally sensitive to *A. cornutum* extract (IC_50_ = 24.15 µg/mL) and *A. cepa* extract (IC_50_ = 24.79 µg/mL), but *A. cepa* extract was slightly more toxic to the HCT116 cell line than *A. cornutum* extract (IC_50_ = 24.73 µg/mL; IC_50_ = 25.97 µg/mL). The cytotoxic effects of both onion extract on the U2OS cell line were considerably lower than those on HeLa and HCT116. The IC_50_ value of the extracts for growth inhibition of U2OS cells was determined to be above 30 µg/mL (IC_50_ = 47.8 µg/mL for *A. cornutum*; IC_50_ = 36.6 µg/mL for *A. cepa*). 

These results are in correlation with a previously reported study concerning good antiproliferative effects *of A. cornutum* and *A. cepa* L. extracts on growth of glioblastoma and breast cancer cell lines. The molecular mechanism of antiproliferative action is probably related with flavonols (Q 3,4′-diglucoside and Q 4′-monoglucoside) found in both extracts. Importantly, the authors showed that *A. cornutum* and *A. cepa* L. extracts did not have an adverse effect on healthy human leukocytes and also proved that both extracts are able to protect DNA from oxidative damage [12]. Similarly, Cho et al. (2016) showed that onion extract can protect human lymphocytes from bleomycin-induced DNA damage [50]. The juice from *Allium macrostemon* bulbs significantly reduced oxidative damage in T-lymphocytes of rats [51]. Özkan et al. (2013) [52] showed that organosulfur rich ethanol extract of *Allium tuncelianum* significantly reduced the chromosomal aberration rate in human lymphocyte cells when compared with the culture treated with a mutagenic compound, mitomycin C (MMC). Millet et al. (2012) [53] showed that fermented aqueous extract (FAE) of *A. cepa* can significantly inhibit HepG2 cancer cell growth. Similar results were obtained with the extracts of other *Allium* species. *Allium flavum* extract showed good antiproliferative activity on a HCT-116 cell line with IC_50_ value < 30 µg/mL, which is in line with the American National Cancer Institute criteria of cytotoxic activity for the crude extracts [54]. In correlation with these results, Simin et al. (2013) [55] showed that active compounds from *A. flavum* are potent inhibitors of growth of cervix epithelioid carcinoma (HeLa), colon adenocarcinoma (HT-29), and breast adenocarcinoma cells (MCF-7). Panduragan et al. (2016) [56] showed that both dry and fresh *Allium ascalonicum* extracts effectively inhibited proliferation of a HepG2 liver cancer cell line. Methanolic flower extract of *A. atroviolaceum* (FAA) showed the ability to inhibit the growth of HeLa cells, both dose- and time- dependent. Importantly, the extract showed no toxicity toward normal 3T3 fibroblast, which demonstrates that FAA might be used as anticancer agent against cervix cancer [57]. The antiproliferative activity of *Allium* species seems to be related with the synergistic effect of various phytochemicals present in their bulbs of leaves, especially flavonoids and sulfur compounds. Two most abundant flavanols in onion species, quercetin 3,4′-diglucoside and quercetin 4′-monoglucoside, have shown the ability to suppress cell proliferation in three cancer cell lines, HepG2, PC-3, and HT-29 [42]. Similarly, the combination of quercetin and kaempferol was able to reduce cell proliferation in human gut (HuTu-80 and Caco-2) and breast cancer cells (PMC42), which is the evidence of the synergistic effect of phytochemicals isolated from different vegetables used in the human diet [58]. Several studies confirmed that volatile sulfur compounds, mostly allyl sulfides, responsible for specific odor of onion species, play an important role in their antiproliferative activity. Dipropyl sulfide (DPS) and dipropyl disulfide (DPDS) isolated from onions are capable to stop carcinogenesis [59]. Other onion sulfur compounds, SAMC, ajoene, methiin, DADS, and DATS, are responsible for strong antiproliferative activity in different cancer cell lines. They exert this effect by inducing the apoptosis [60,61,62]. Similarly, DATS, a major organosulfur compound isolated from garlic, can inhibit cell proliferation through the cell cycle arrest or the induction of apoptosis [63]. DATS showed high toxicity toward prostate cancer cells [64], while DADS induced apoptosis in HL-60, HCT-15, and neuroblastoma cells [65,66,67]. It also induced apoptosis in COLO 25 cell line by promoting caspase-3 activity [68]. Provided research studies revealed that organosulfur compounds, together with flavonoids, exhibit the highest antiproliferative activity, and thus can be used to reduce the risk of cancer development. 

Since HeLa cells showed the highest sensitivity to *Allium* extracts among all tested cancer cell lines, we further wanted to investigate the possible mechanism of proliferative activity by using the DNA fragmentation assay, DAPI staining, and qPCR analysis.

### 2.4. DNA Fragmentation Analysis of HeLa Cells

**Figure 2:**
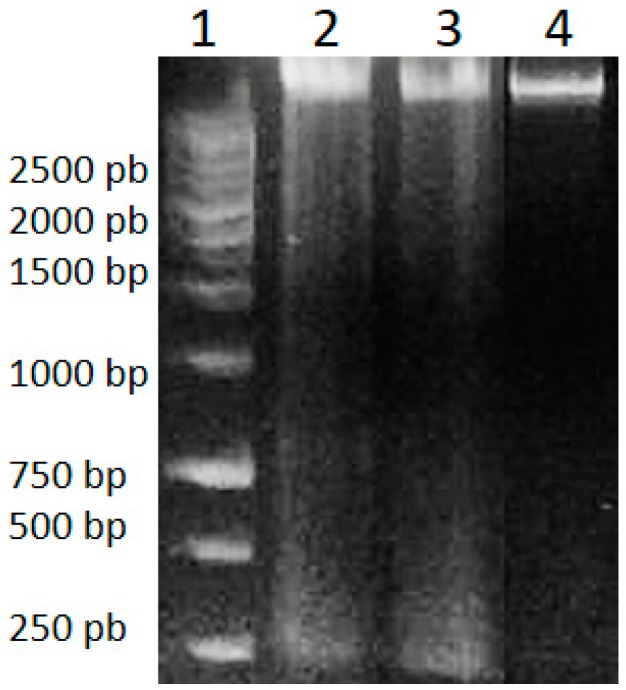
DNA Ladder (**1**), genomic DNA of HeLa cells treated with *A. cornutum* extract (**2**), genomic DNA of HeLa cells treated with *A. cepa* extract (**3**), genomic DNA of untreated HeLa cells (**4**).

Genomic DNA isolated from HeLa cells after 48 h of treatment with *A. cornutum* and *A. cepa* extracts was found to be fragmented, in contrast to untreated cells, which showed intact DNA on gel electrophoresis (Figure 2). DNA fragmentation is considered to be a molecular hallmark of apoptosis [69], resulting from the cleavage of DNA into fragments of ~200 bp, by the activation of CAD (caspase-activated DNase) cascade of caspases. The appearance of DNA fragmentation in treated DNA samples in comparison with the intact DNA in untreated cells confirmed the induction of apoptosis by *Allium* extracts.

### 2.5. Morphological Changes of HeLa Cells after Treatment with Onion Extracts

**Figure 3:**
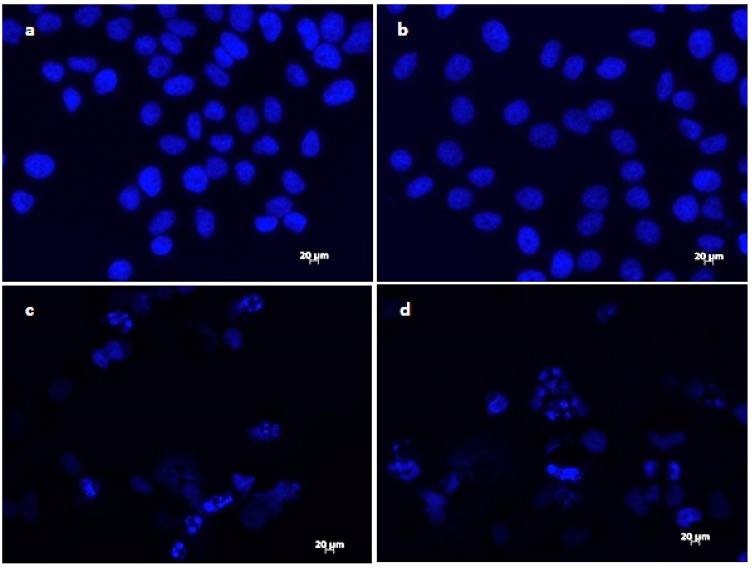

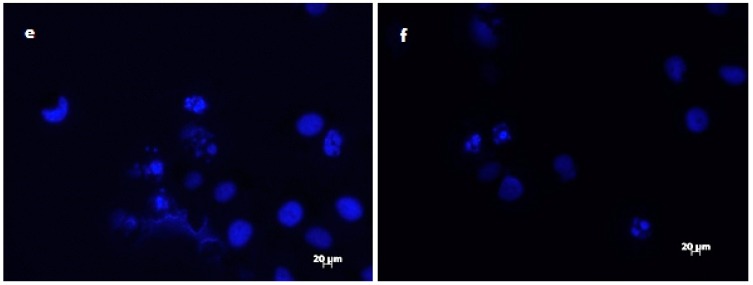
Morphological changes in HeLa cells treated with onion extracts examined by DAPI staining. Control (untreated cells) (**a**,**b**), HeLa cells treated with *A. cornutum* extract (IC_50_ = 24.15 µg/mL) (**c**,**d**), and HeLa cells treated with *A. cepa* L. extract (IC_50_ = 24.79 µg/mL) (**e**,**f**).

The results of DAPI staining analysis confirmed that treatment with onion extracts at IC_50_ concentration induced apoptosis in HeLa cells. As illustrated in Figure 3a,b, untreated cells are round with normal morphology. Cells treated with onion extracts (Figure 3c–e) showed changed morphology, cell shrinkage, chromatin condensation, fragmentation of DNA, and formation of apoptotic bodies [70]. These changes are an indicator of cell death and, therefore, the evidence of possible mechanism of cytotoxic activity of both extracts.

### 2.6. Gene Expression Analysis in HeLa Cells

**Figure 4:**
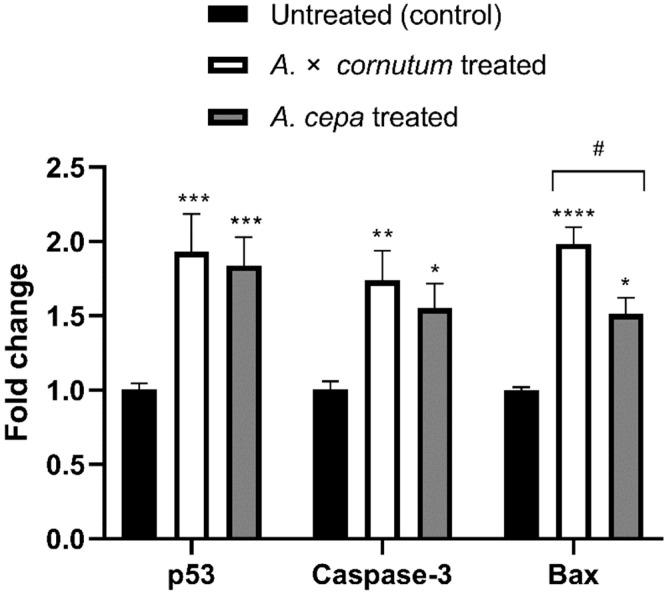
Effects of *A. cornutum* and *A. cepa* L. extracts on p53, Caspase-3, and Bax mRNA expression in treated HeLa cells. The relative quantification was performed by delta delta Ct method, and the levels of gene expression were normalized with GAPDH housekeeping gene. The statistical significance was assessed by two-way ANOVA, followed by Dunnett’s multiple-comparisons test. Columns with bars represent mean ± SD, and significant levels compared to control are marked as * *p* < 0.05; ** *p* < 0.01, *** *p* < 0.001, and **** *p* < 0.0001 (Data S5); significant levels compared to *A. cornutum* with *A. cepa* are marked as ^#^ *p* < 0.05 (Data S6).

To investigate whether the cytotoxic activity of *Allium* extracts was guided by the apoptosis, we measured the mRNA level of three genes involved in apoptosis and cell cycle, p53, Caspase-3, and Bax. As shown in Figure 4, the expression of p53, Caspase-3, and Bax genes was significantly upregulated in treated HeLa cells in comparison to untreated.

Apoptosis is a programmed cell death that can be activated by different stimuli which induce DNA damage [71]. There are two major apoptotic pathways, extrinsic and intrinsic mitochondrial, both leading to the activation of caspases. The morphological features of apoptosis are characterized by cell shrinkage, membrane breakage, DNA fragmentation, and formation of apoptotic bodies [72]. The results of DNA fragmentation test and DAPI staining illustrated all the typical morphological changes of apoptosis in treated HeLa cells. In addition, qPCR results showed the increased expression of the genes related with apoptosis in HeLa treated cells, indicating that apoptosis was trigged by the administration of the extracts of both *Allium* species. Polyphenols showed to be able to induce the oxidative stress in cancer cells, leading to cell cycle disruption, DNA fragmentation, and finally apoptosis [73,74]. Numerous experiments suggested that organosulfur and phenolic compounds isolated from *Allium* species could induce apoptosis in cancer cells by the induction of p53 gene and activation of Caspase-3 [57,66,75]. The tumor-suppressor gene p53, which is often called “guardian of the genome”, is involved in the regulation of cell cycle, apoptosis, and DNA repair. Wu and Shen (2018) [76] suggested the role of p53 gene in activation of Bax in cancer cells. Similarly, we found that *Allium* extracts upregulated the expression of p53, Bax, and Caspase-3 genes in statistically significant manner (Figure 4). Upregulation of p53 activates pro-apoptotic genes, such as Bax, resulting in the permeability of mitochondrial membrane and releasing of cytochrome c, which activates initiator caspase, Caspase-9. Caspase-9 cleaves and activates the downstream effector caspase, Caspase-3, which generates the caspase-cascade reaction and intrinsic mitochondrial cell death [77,78]. The results of this study suggest that increased mRNA expression of p53 could be associated with upregulation of Bax and activation of Caspase-3, indicating that mitochondrial apoptotic pathway might be involved in the apoptosis induction by onion extracts in the HeLa cell line. Therefore, further studies on protein level must be conducted to confirm the mechanism of apoptosis occurring in treated HeLa cells.

## 3. Materials and Methods

### 3.1. Plant Material and Preparation of Extracts

*Allium cornutum* Clementi ex Visiani, 1842, was obtained from the local gardens along the Dalmatian Coast, and *Allium cepa* L. was purchased at the local market with domestic products. Onion extracts were prepared, and HPLC analysis was performed according to Fredotović et al. (2017) [12], as follows: homogenized dry bulbs were extracted, using 70% methanol water (*v/v*; 100 mL). After 30 min of extraction, the extract was centrifuged at 3000 rpm for 15 min. Pooled supernatants were dried under vacuum, using a rotary evaporator. Samples were dried in a vacuum oven until they reached constant weight. The dry residues were dissolved in 10% DMSO and filtered through a 0.45 µm nylon filter disc. HPLC analysis of the extracts was performed in order to identify main flavonols and anthocyanins. The most abundant flavonols found in both onions were quercetin 3,4′-diglucoside and quercetin 4′-monoglucoside. Three anthocyanins were found in both extracts: peonidin glucoside, petunidin glucoside, and malvidin glucoside. The fourth anthocyanin, delphinidin glucoside, was found only in *A. cornutum* extract [12].

### 3.2. Extraction and Analysis of Volatile Sulfur Compounds

The isolation of headspace volatiles was performed by using a manual SPME fiber with the layer of carboxen/polydimethylsiloxane (CAR/PDMS). For HS-SPME extraction, 1 g of the sample was placed in a 15 mL glass vial, and the vial was maintained in the water bath at 40 °C, during the equilibration period (15 min) and the extraction period (40 min). After the sampling, the SPME fiber was withdrawn into a needle, removed from the vial, and inserted into injector for 7 min, where the extracted volatiles were thermally desorbed directly to the GC column. The samples were analyzed by GC–MS system (Agillent Technologies GC 7820A and MSD 5977E, Palo Alto, CA, USA). Operating conditions were as follows: column HP-5MS ((5%-phenyl)-methylpolysiloxane), 30 m × 0.25 mm i.d., coating thickness 0.25 μm) and column temperature was programmed at 70 °C isothermal for 2 min, and then increased to 200 °C at a rate of 3 °C/min and held isothermal for 15–18 min; helium as the carrier gas at 1 mL/min; injector temperature 250 °C; the split ratio 1:50; ionization voltage 70 eV; ion source temperature 230 °C; the mass range at *m*/*z* 30–350. The individual peaks were identified by comparison of their retention indices (relative to C_9_-C_25_ *n*-alkanes) to available authentic samples and literature data [79], as well as by comparison of the mass spectra with the Wiley 9 MS library (Wiley, New York, USA) and NIST 14 (Gaithersburg, Germany) databases. The percentage composition was calculated by using the normalization method (without correction factors) from the GC peak areas. The HS-SPME/GC–MS analyses were performed in triplicate.

### 3.3. Analysis of Amino Acid Composition

Amino acids were separated by reverse-phase HPLC, using a Perkin Elmer Series 200 LC system and TotalChrom Workstation software, Waters AccQ•Tag™ derivatization chemistry, AccQ™•Tag-column, and fluorescence detection. Before the analysis, the onion samples were crushed in liquid nitrogen and hydrolyzed as follows: 10 mg of sample was mixed with 1 mL of HCl/H_2_O (50:50, *v/v*) and heated overnight in oven at 105–110 °C. After cooling at room temperature, the solution was neutralized by adding solid NaHCO_3_. The resulting hydrolyzed samples were then subjected to derivatization. In the glass sample tube (6 × 50 mm), 70 µL of AccQ•Tag™ Borate Buffer was added to 10 µL neutralized hydrolysate, and vortexed briefly. Furthermore, 20 µL of AccQ•Tag™ Fluor Reagent was also added and vortexed. The solution was left to stand for a minute, at room temperature. The solution was transferred in a vial with limited volume insert, caped, and heated for 10 min at 55 °C. The volume of 5 µL of derivatized sample was injected in the HPLC system. The separation was achieved on the column AccQ•TagTM 3.9 × 150 mm designed for hydrolysate amino acid analysis and Nova-Pak C18 guard column (Waters). The oven temperature was set at 37 °C, and elution solvent A was AccQ•Tag TM concentrate diluted with water. The solvent B was a mixture of acetonitrile and water (60:40, *v*/*v*). The flow rate of the mobile phase was 1 mL/min. A gradient separation was achieved by using a pump program as follows: 0–0.5 min 100% A, 0.5–15.5 min 93% A, 15.5–19.5 min 89% A, 19.5–32.5 min 67% A, 32.5–33.5 min 67% A, 33.5–34.5 min 0% A, 34.5–43.0 min 0% A, 43.0–44.0 min 100% A, and 44.0–50.0 min 100% A. The amino acids were detected by a fluorescence detector, using the excitation wavelength at 250 nm and emission at 395 nm. Amino acids were identified and quantified by using the Waters Amino Acid Hydrolysate Standard according to the Waters AccQ•Tag Chemistry Package Instruction Manual. The identification was carried out by comparing the retention time of the sample with pure standards, while the quantification was carried out by building the calibration curve based on five different standard concentrations for each amino acid. Retention time (RT), limit of detection (LOD), limit of quantification (LOQ), and coefficient of correlation (R^2^) for amino acids are presented as Supplementary file (Data S7).

### 3.4. Cell Proliferation Assay

Cytotoxicity of *Allium* extracts was determined on three cancer cell lines: cervical cancer cell line (HeLa), human colon cancer cell line (HCT116), and human osteosarcoma cell line (U2OS). The cells were a kind gift from Janoš Terzić laboratory at the School of Medicine, University of Split. The cytotoxicity of the extracts was determined by using the MTS-based CellTiter 96^®^ Aqueous assay (Promega). Cells were grown in an incubator at 37 °C and 5% CO_2_, until they reached 80% confluency. Cells were counted with a handheld automated cell counter (Scepter, Merck), and 5000 cells/well were seeded in 96-well plates containing a serial dilution of both extracts. Cells were further grown for an additional 48 h, after which 20 µL of MTS tetrazolium reagent (Promega) was added to each well. DMSO was used as vehicle control. After 3 h of incubation, the absorbance at 490 nm was measured by a 96-well plate reader (Bio-Tek, EL808). The measurements were performed in quadruplets, and IC_50_ values were calculated from three independent experiments, using GraFit 6 data analysis software (Erithacus, East Grinstead, UK).

### 3.5. DNA Fragmentation Assay

HeLa cells were seeded in 6-well plate. After overnight incubation, medium was replaced with the fresh one, and IC_50_ doses of *A. cornutum* and *A. cepa* extracts were added. After 24 h, cells were harvested by trypsinization, washed with PBS, and counted. Next, 5 × 10^5^ cells were transferred in an Eppendorf tube and centrifuged, and pellet was treated with lysis buffer (10 mM Tris (pH 8), 10 mM EDTA, and 0.5% Triton X-100) for 2 h at 37 °C. Genomic DNA was incubated with RNase A (20 µg/mL) for 1 h at 37 °C and then with proteinase K (50 µg/mL) for 2 h at 50 °C. After extraction with phenol:chloroform:isoamyl alcohol (25:24:1), DNA was washed with 70% ethanol, air-dried, dissolved in distilled water, and separated in 1.5% agarose gel containing EtBr (10 mg/mL). The DNA fragmentation pattern was visualized under UV transilluminator and photographed by using a gel documentation system.

### 3.6. DAPI Staining

DAPI is a fluorescent dye that intercalates into the AT-rich heterochromatin regions of DNA (also binds to RNA, but significantly weaker). Since DAPI can pass through the intact cell membrane, it can be used to stain live and fixed cells. Changes in the nuclear morphology, as a consequence of apoptosis, can be easily observed by staining with DAPI fluorescent dye. Healthy cells show a diffusely stained nuclei, while cells in apoptosis may have several specific patterns, such as condensed chromatin, nuclear fragmentation, or a significant decrease in the cell nuclei. Cells were plated in 6-well plate at a density of 1 × 10^5^ cells/well. After 48 hours of treatment with *A. cornutum* and *A. cepa* extracts (IC_50_ doses), cells were washed with PBS and fixed in 4% paraformaldehyde solution for 10 minutes, at room temperature. After being washed in PBS, cells were permeabilized with buffer containing 3% paraformaldehyde and 0.5% Triton X-100 and stained with DAPI. The nuclear morphology of the cells was observed under a fluorescent microscope (Zeiss Axioimager M1 epifluorescence microscope with high-resolution CCD camera, Vienna, Austria) using Axio Vision rel. 4.7 software (Carl Zeiss, Vienna, Austria).

### 3.7. Real-Time PCR Analysis

HeLa cells were plated and treated with onion extracts, as previously described. Total RNA was isolated by using TRI reagent (Sigma, St. Louis, MO, USA). Next, 1 µg of RNA was reverse transcribed to cDNA, using the Superscript Frist Strand III kit (Thermo Fisher, Invitrogen, Waltham, MA, USA). Real-time PCR reaction was performed by using the ABI 7500 Real-Time PCR System (Applied Biosystem, Foster City, CA, USA) with PCR master mix containing SYBR Green (Thermo Fisher, Invitrogen, Waltham, MA, USA), under the following reaction conditions: 95 °C for 5 min, 40 cycles of denaturation at 95 °C for 10 s, annealing and synthesis at 60 °C for 10 s, and final extension at 72 °C for 10 s. For relative quantification, all samples were normalized to GAPDH housekeeping gene. Melting curves were constructed immediately after amplification, to verify the specificity of PCR products. Relative gene expression was calculated by using the 2^−ΔΔCt^ method. The primer sequences used in this study are presented in Table 3.

### 3.8. Statistical Analysis

Statistical analysis was performed by using GraphPad Prism Version 8. All data are expressed as mean ± SEM (*n* ≥ 3). The statistical significance was assessed by two-way ANOVA, followed by Dunnett’s multiple-comparisons test and Sidak’s multiple-comparisons test. Differences were considered as * *p* < 0.05 (^#^ *p* < 0.05); ** *p* < 0.01; and *** *p* < 0.001.

## 4. Conclusions

*Allium* species, used in our study, proved to be a good source of phytochemicals, especially organosulfur compounds, which play a crucial role in preventing proliferation of cancer cells. Dipropyl disulfide, diisopropyl trisulfide, 1-propanethiol, and (Z)-prop-1-enyl propyl trisulfide are the most dominant volatile sulfur compounds in both onion species, with higher abundance in *A. cornutum*. The higher abundance in triploid hybrid onion can be explained by the higher number of cysteine sulfoxides due to the hybridization of common onion and wild *Allium* species.

Arginine (Arg) and glutamic acid (Glu) are the main free amino acids found in *A. cornutum* and *A. cepa*. They play a crucial role as nitrogen reservoirs during onion maturation and might be responsible for the color of onion seeds. Glu is also important “umami” substrate responsible for specific onion taste.

Both onion extracts inhibited the growth of HeLa, HCT116, and U2OS cells. In addition, transcriptional activation of p53, Bax, and Caspase-3 genes in HeLa cells is stimulated by the administration of onion extracts. The morphological changes, such as cell shrinkage, fragmentation of nuclei, and formation of apoptotic bodies, are visible evidence of apoptosis occurring in treated HeLa cells. As for the triploid onion, *A. cornutum*, this was the first study to report its organosulfur and amino acid content. The present study demonstrated that *A. cornutum* and *A. cepa* extracts can potentially be used against cervical carcinoma. However, protein expression analysis, in vivo studies after the oral administration of onion extracts, and clinical trials need to be done to evaluate their possible use as natural chemotherapeutic agents against human cervical cancer, as well as with other cancer cell lines.

## Figures and Tables

**Table 1:** Headspace-volatile compounds form freshly cut bulbs of *Allium* species.

No.	Compound	RI	*A. cornutum* (Area %) ± SD	*A. cepa* L. (Area %) ± SD
1	Sulfur dioxide	<900	0.3 ± 0.01	0.4 ± 0.02
2	Methanethiol	<900	0.9 ± 0.04	0.8 ± 0.03
3	Propanal	<900	0.2 ± 0.01	0.2 ± 0.01
4	Carbon disulfide	<900	0.5 ± 0.02 ^a^	0.2 ± 0.01 ^b^
5	Prop-2-en-1-thiol	<900	0.3 ± 0.02	0.3 ± 0.03
6	1-Propanethiol	<900	14.1 ± 0.20	13.1 ± 0.31
7	Dimethyl disulfide	<900	0.1 ± 0.01	0.0 ± 0.00
8	2-Methylpentanal	<900	0.1 ± 0.01	0.1 ± 0.01
9	(*E*)-Hex-2-enal	<900	0.3 ± 0.02	0.2 ± 0.01
10	(*Z*)-Hex-3-en-1-ol	<900	0.2 ± 0.01	0.2 ± 0.01
11	Propyl hydrosulfide	<900	0.3 ± 0.02	0.4 ± 0.03
12	(*E*)-Hex-3-en-1-ol	<900	0.9 ± 0.04 ^a^	0.5 ± 0.02 ^b^
13	S-Propyl ethanetihoate	<900	1.5 ± 0.10	1.3 ± 0.15
14	2,5-Dimethylthiophene **	912	0.1 ± 0.01 ^b^	0.2 ± 0.01 ^a^
15	Methyl propyl disulfide	938	0.8 ± 0.01 ^b^	0.9 ± 0.01 ^a^
16	Methyl (*E*)-prop-1-enyl disulfide	947	0.0 ± 0.00 ^b^	0.1 ± 0.01 ^a^
17	Ally propyl disulfide	1095	0.6 ± 0.01 ^b^	0.9 ± 0.01 ^a^
18	Dipropyl disulfide	1111	45.5 ± 0.01 ^a^	31.9 ± 0.01 ^b^
19	(*E*)-prop-1-enyl propyl disulfide	1119	0.1 ± 0.01 ^b^	3.8 ± 0.20 ^a^
20	Methyl methylthiomethyl disulfide	1130	2.8 ± 0.10 ^a^	0.5 ± 0.04 ^b^
21	Methyl propyl trisulfide	1155	0.2 ± 0.01	0.2 ± 0.01
22	Diisopropyl trisulfide	1328	18.3 ± 0.21 ^b^	24.6 ± 0.41 ^a^
23	(*Z*)-Prop-1-enyl propyl trisulfide	1336	3.1 ± 0.11	2.8 ± 0.10
24	(*E*)-Prop-1-enyl propyl trisulfide	1339	0.7 ± 0.05	1.7 ± 0.10
25	Dipropyl tetrasulfide	1565	0.7 ± 0.02 ^a^	0.0 ± 0.00 ^b^
26	*cis*-3,6-Diethyl-1,2,4,5-tetrathiane *	1582	0.4 ± 0.01 ^b^	1.7 ± 0.12 ^a^
27	*trans*-3,6-Diethyl-1,2,4,5-tetrathiane *	1584	1.8 ± 0.10 ^b^	4.8 ± 0.21 ^a^

RI = retention indices relative to C_9_–C_25_ alkanes; * = tentatively identified; ** = correct isomer is not identified; SD = standard deviation of triplicate analysis; significant differences were determined using two-way ANOVA, followed by Sidak’s multiple-comparisons test. ^a, b^ Mean values with different superscript letters indicate a statistically significant difference between two onions (*p* < 0.05) (Data S1).

**Table 2:** Amino acid composition of triploid hybrid onion *A. cornutum* and common onion *A. cepa*.

Amino Acids	*A. cornutum* (mg/g DW) * ± SD	*A. cepa* L. (mg/g DW) * ± SD
Asp (Aspartic acid)	4.941 ± 0.043 ^b^	6.100 ± 0.083 ^a^
Ser (Serine)	2.921 ± 0.004	2.846 ± 0.031
Glu (Glutamic acid)	14.964 ± 0.008 ^a^	11.238 ± 0.096 ^b^
Gly (Glycine)	2.042 ± 0.013	2.217 ± 0.015
His (Histidine)	1.758 ± 0.015	1.667 ± 0.052
Arg (Arginine)	16.492 ± 0.256 ^a^	12.213 ± 0.094 ^b^
Thr (Threonine)	3.324 ± 0.013 ^b^	4.003 ± 0.076 ^a^
Ala (Alanine)	1.675 ± 0.002 ^b^	2.027 ± 0.023 ^a^
Pro (Proline)	1.434 ± 0.024 ^b^	1.702 ± 0.128 ^a^
Cys (Cysteine)	0.144 ± 0.002	0.023 ± 0.005
Tyr (Tyrosine)	1.719 ± 0.010 ^b^	2.277 ± 0.049 ^a^
Val (Valine)	2.005 ± 0.003 ^b^	2.375 ± 0.072 ^a^
Met (Methionine)	0.013 ± 0.005	0.010 ± 0.005
Lys (Lysine)	2.322 ± 0.010	2.328 ± 0.018
Ileu (Isoleucine)	1.426 ± 0.006 ^b^	1.818 ± 0.007 ^a^
Leu (Leucine)	2.284 ± 0.014 ^b^	2.949 ± 0.026 ^a^
Phe (Phenylalanine)	2.198 ± 0.014	2.378 ± 0.006

Significant differences were determined by using two-way ANOVA, followed by Sidak’s multiple-comparisons test. * Values are means ± SD (standard deviations) of triplicate determinations; ^a, b^ mean values with different superscript letters indicate a statistically significant difference between two onions (*p* < 0.05) (Data S3).

**Table 3:** Primers used in real-time PCR reaction.

Gene	Primer	Sequence (5′-3′)
*GAPDH*	Forward	TCGACAGTCAGCCGCATCTT
	Reverse	GCCCAATACGACCAAATCCGT
p53	Forward	GCTCACTCCAGCCACCTGAA
	Reverse	CCAAAATGGCAGGGGAGGGA
Caspase-3	Forward	CATACTCCACAGCACCTGGTTA
	Reverse	CAGCATGGCACAAAGCGACT
Bax	Forward	CAAACTGGTGCTCAAGGCCC
	Reverse	GCACTCCCGCCACAAAGATG

## Supplementary Materials

The following are available online at https://www.mdpi.com/2223-7747/9/1/98/s1. Data S1: Statistical analysis of headspace volatiles. Data S2: Table S3: Volatile sulfur compounds of several *Allium* species. Data S3: Statistical analysis of amino acids. Data S4: Statistical analysis of antiproliferative activity. Data S5: Statistical analysis of gene expression 1. Data S6: Statistical analysis of gene expression 2. Data S7: Table S4: Retention time (RT), limit of detection (LOD), limit of quantification (LOQ), and coefficient of determination (R^2^) for amino acids.
