# Supplementary material for: Comparison of Organosulfur and Amino Acid Composition between Triploid Onion Allium cornutum Clementi ex Visiani, 1842, and Common Onion Allium cepa L., and Evidences for Antiproliferative Activity of Their Extracts
Source: Plants (Basel). 2020 Jan 13;9(1):98. doi: 10.3390/plants9010098 (PMC7020437; doi:10.3390/plants9010098)
Supplement: Supplementary file 1 [file plants-09-00098-s001.zip › plants-677263-supplementary-1/Data S2_Table 3_Volatile sulfur compounds of several Allium species.docx]

**Table S3.** Volatile sulfur compounds of several *Allium* species

| **Sample** | **Volatile sulfur compounds** | | **Extraction method** | | | **Identification method** | **Reference** | | |  |
| --- | --- | --- | --- | --- | --- | --- | --- | --- | --- | --- |
| *Allium hookeri (*roots) | | | Ethylsulfide, Dimethylsulfide, 3,3-Thiobis-1-propene, n-Propylcis-1-propenylsulfide, Diethyldisulfide, Methyl-trans-propenyl-disulfide, Methylpropyldisulfide, Trans-propenylmethyldisulfide, Dimethyltrisulfide, Propenesulfide, Diallyldisulfide, Butylpropenylsulfide , (E,E)-Bis(1-propenyl)disulfide, trans-Propenylpropyldisulfide, 2-Propenylpropyldisulfide, Methylmethylthiomethyldisulfide, Methylpropyltrisulfide, Dimethyltetrasulfide, Diallyltrisulfide, Dipropyltrisulfide, Methyl 2-propenyltetrasulfide, Dimethyltrisulfide, Di-2-propenyltetrasulfide | | simultaneous steam distillation-solvent extraction | Gas Chromatography-Olfactometry (GC/MS-O) | | [80] | |  |
| *Allium ursinum (*essential oil) | | | Allylthiol, Methyl hydrogen disulﬁde, Allyl methyl sulﬁde, Dimethyl disulﬁde, Methyl propyl sulfoxide, Diallyl sulﬁde, Allyl propyl sulﬁde, 2,4-Dimethylthiophene, 3-(Methylthio) propanal (syn. methional), Allyl propenyl sulﬁde, 3,4-Dimethylthiophene, Propenyl propyl sulﬁde. Allyl methyl disulﬁde, Methyl pentyl sulﬁde, Methyl (Z)-1-propenyl disulﬁde and methyl propyl disulﬁde, 3-(Methylthio)butanal, Methyl (E)-1-propenyl disulﬁde, Dimethyl trisulﬁde, 3-Thiophenethiol, 3-Thiophenecarboxaldehyde, 2-Thiophenecarboxaldehyde, Hexyl methyl sulﬁde, Methyl butanedithioate, Diallyl disulﬁde, (E)-2-(Methylthiomethyl)but-2-enal, Allyl propyl disulﬁde and allyl (Z)-1-propenyl disulﬁde, Allyl (E)-1-propenyl disulﬁde, Dipropyl disulﬁde and (Z)-1-propenyl propyl disulﬁde, Methyl 1-(methylthio)methyl disulﬁde and (E)-1-propenyl propyl disulﬁde, (Z,Z)-Bis(1-propenyl) disulﬁde, Heptyl methyl sulﬁde, (E,E)-Bis(1-propenyl) disulﬁde, Allyl methyl trisulﬁde, Methyl propyl trisulﬁde, Methyl (Z)-1-propenyl trisulﬁde, Methyl (E)-1-propenyl trisulﬁde, Methyl 1-(methylthio)ethyl disulﬁde, 2-Mercapto-3,4-dimethyl-2,3-dihydrothiophene, Allyl hexyl sulﬁde, Dimethyl tetrasulﬁde, 3-Methyl-5-ethyl-1,2,4-trithiolane, Methyl octyl sulﬁde, Hexyl methyl disulﬁde, Methyl (methylthio)propyl disulﬁde, 2-Hexylthiophene, Propyl (propylthio)methyl sulﬁde, Allyl (methylthio)methyl disulﬁde, Diallyl trisulﬁde, (Methylthio)methyl propyl disulﬁde, (Methylthio)methyl (Z)-1-propenyl disulﬁde, (Methylthio)methyl (E)-1-propenyl disulﬁde, Allyl propyl trisulﬁde, Allyl (Z)-1-propenyl trisulﬁde, Allyl (E)-1-propenyl trisulﬁde, Dipropyl trisulﬁde, Allyl 1-(methylthio)ethyl disulﬁde, (Z)-1-Propenyl propyl trisulﬁde, (E)-1-Propenyl propyl trisulﬁde and (Z,Z)-Bis(1-propenyl) trisulﬁde, (E,E)-Bis(1-propenyl) trisulﬁde, 5,6-Dimethyl-2,3,7-trithiabicyclo[2.2.1]heptane, Allyl methyl tetrasulﬁde, Methyl propyl tetrasulﬁde, Allyl octyl sulﬁde, Methyl 1-propenyl tetrasulﬁde, Octyl propyl sulﬁde, Allyl 1-(methylthio)propyl disulﬁde, 1-(Methylthio)propyl propyl disulﬁde, Methyl 1-(1-propenylthio)propyl disulﬁde, 1-(Methylthio)propyl 1-propenyl disulﬁde, 2,3-Dimethyl-5,6-dithiabicyclo[2.1.1]hexane 5,5-dioxide, Dimethyl pentasulﬁde, Methyl 1-(methylthio)propyl trisulﬁde, Methyl 3,4-dimethyl-2-thienyl disulﬁde, Diallyl tetrasulﬁde, Allyl 1-propenyl tetrasulﬁde, (Z)-1-Propenyl propyl tetrasulﬁde, (E)-1-Propenyl propyl tetrasulﬁde, 2,4-Dimethyl-5,6-dithia-2,7-nonadienal, Sulfur (S8) | | hydrodistillation | Gas Chromatography – Flame Ionization Detector (GC/FID), Gas chromatography–mass spectrometry (GC/MS) combined with quantitative structure-property relationship (QSPR) | | | [81] | |
| *Allium schoenoprasum* L. (bulbs) | | | Methyl propyl trisulfide, 1,5-Dithiocane, cis-Propenyl propyl trisulfide, 2-Methyl-2-methylthio-1-propanol, Trisulfide dimethyl, trans-Propenyl propyl trisulfide, Trisulfide dipropyl, 1,4-Dimethyl tetrasulfane, 2,4-Dimethyl-5,6-dithia-2,7-nonadienal, Disulfide methyl 1-(methylthio)propyl, 1-Propene, 1-(methylthio)-, (z)-, 1,3-Dithiane, 1,2,3-Trithiolane, 4-methyl-, 1-Propene 1-(methylthio)-, (e)-, Disulfide methyl propyl, Disulfide dipropyl, 3-Ethyl-5-methyl-1,2,4-trithiolane, 6-Ethyl-4,5,7,8-tetrathianonane, Methyl 1-propenyl disulfide, (z)-, 1-Propyl-2-(4-thiohept-2-en-5-yl) disulfide, Methyl 1-propenyl disulfide, (e)-, Disulfide dimethyl, 1,2-Dithiolane, 4,6-Diethyl-1,2,3,5-tetrathiolane, Thiophene, 2,4-dimethyl-, Trisulfide, methyl 2-propenyl, 2-Mercapto-3,4-dimethyl-2,3-dihydrothiophene, 8-Ethyl-4,5,6,7,9-pentathiadecane, Carboisopropoxy methoxy sulfide, methyl 1-(methylthio)ethyl, Diallyl disulphide, Carboisopropoxy carbomethoxymethyl disulfide, 6-ethyl-4,5,7,8-tetrathia-2-nonene, Methyl methanesulfonylacetate, Disulfide methyl 2-propenyl | | hydrodistillation | Gas Chromatography combined with time-of-flight mass spectrometry detector (GC/MS-TOF) | | | [82] | |
| *Allium tuberosum and chinese* (leaves) | | | ethyl acetate, ethyl propionate, dimethyl disulfide, ethyl cis-1-propenyl sulfide, ethyl methyl disulfide, diallyl sulfide, allyl methyl disulfide, methyl propyl disulfide, methyl cis-1-propenyl disulfide, dimethyl trisulfide, ethyl propyl disulfide, ethyl cis-1-propenyl disulfide, ethyl trans-1-propenyl disulfide, butyl methyl disulfide, limonene, ethyl methyl trisulfide, diallyl disulfide, allyl cis-1-propenyl disulfide, propyl cis-1-propenyl disulfide, allyl trans-1-propenyl disulfide, dipropyl disulfide, propyl trans-1-propenyl disulfide, allyl methyl trisulfide, methyl propyl trisulfide, methyl cis-1-propenyl trisulfide, methyl trans-1-propenyl trisulfide, dimethyl tetrasulfide, butyl methyl trisulfide, diallyl trisulfide, propyl cis-1-propenyl trisulfide, dipropyl trisulfide, propyl trans-1-propenyl trisulfide, propyl cis-l-propenyl tetrasulfide, methyl pentyl tetrasulfide, propyl trans-propenyl tetrasulfide, dipropyl tetrasulfide, allyl propyl tetrasulfide, propyl methyl pentasulfide, 2,3-dihydro-2n-octyl-5-methylfuran-3-one, propyl cis-l-propenyl pentasulfide | | simultaneous steam distillation-solvent extraction | Gas chromatography–mass spectrometry (GC/MS) | | | [83] | |
| *Allium sativum* L. (essential oil) | | | Dimethyl disulﬁde, 2-Methyl-4-pentenal, 2-Methylene-4-pentenal, 3-Methylthiophene, Hexanal, 1,2-Dithiolane, Diallyl sulﬁde, Allyl propyl sulﬁde, Allyl (Z)-1-propenyl sulﬁde, Allyl (E)-1-propenyl sulﬁde, 3,4-Dimethylthiophene, Allyl methyl disulﬁde, Methyl (Z)-1-propenyl disulﬁde, Methyl (E)-1-propenyl disulﬁde, 1,2-Dithiolene, Dimethyl trisulﬁde, Diallyl disulﬁde, Allyl (Z)-1-propenyl disulﬁde, Allyl (E)-1-propenyl disulﬁde, Allyl methyl trisulﬁde, Methyl propyl trisulﬁde, 4-Methyl-1,2,3-trithiolane, Methyl (Z)-1-propenyl trisulﬁde, Methyl (E)-1-propenyl trisulﬁde, 3-Vinyl-4H-1,2-dithiine, 1,2,3-Trithia-4-cyclohexene,Allicin, 2-Vinyl-4H-1,3-dithiine, Methyl (methylsulﬁnyl)methyl sulﬁde, Diallyl trisulﬁde, Allyl propyl trisulﬁde, Allyl (E)-1-propenyl trisulﬁde, 5-Methyl-1,2,3,4, Unidentiﬁed, 1,4-Dihydro-2,3-benzoxathiin 3-oxide, [(E)-1-Propenyl] 2-thiopent-3-yl disulﬁde, Diallyl tetrasulﬁde, Propyl 4-thiohept-2-en-5-yl disulﬁde, 4-Methyl-1,2,3,5,6-pentathiepane, Cyclooctasulfur | | laboratory hydrodistillation, industrial hydrodistillation, and industrial steam distillation | Gas chromatography–mass spectrometry (GC/MS) | | | [84] | |
| *Allium vineale* (essential oil) | | | 2-Furaldehyde, (2E)-Hexenal, (3Z)-Hexenol, 2,4-Dimethylthiophene, Allyl methyl disulﬁde, Methyl (Z)-1-propenyl disulﬁde, Methyl (E)-1-propenyl disulﬁde, Benzaldehyde, Dimethyl trisulﬁde, Diallyl disulﬁde, Allyl (Z)-1-propenyl disulﬁde, Allyl (E)-1-propenyl disulﬁde, 1-Propenyl propyl disulﬁde, Methyl methylthiomethyl disulﬁde, Allyl methyl trisulﬁde, 4-Methyl-1,2,3-trithiolane, Methyl propyl trisulﬁde, Methyl (Z)-1-propenyl trisulﬁde, Methyl (E)-1-propenyl trisulﬁde, Dimethyl tetrasulﬁde, Allyl methylthiomethyl disulﬁde, Diallyl trisulﬁde, Allyl (Z)-1-propenyl trisulﬁde, p-Vinylguaiacol, Allyl propyl trisulﬁde, 5-Methyl-1,2,3,4, Methyl methylthiomethyl trisulﬁde, Allyl methyl tetrasulﬁde, Allyl methylthiomethyl trisulﬁde Unidentiﬁed, 4-Methyl-1,2,3,5,6-pentathiepane | | hydrodistillation | Gas chromatography–mass spectrometry (GC/MS) | | | [84] | |
| *Allium hirtifolium* Boiss (bulbs and seeds) | | | Disulfide, dimethyl, Dimethyl trisulfide,  2,3,5-trithiahexane, Chloromethyl methyl sulfide, N-butyl-Benzene sulfonamide, 2,4-Dithiapentane, 4-Mercaptopyridine | | headspace method | headspace- Gas chromatography-Mass Spectrophotometry (GC/MS) | | | [85] | |
| *Allium ampeloprasum* Var. Bulga (freshly cut) | | | 1-propanethiol, methyl propyl disulfide, methyl propenyl disulfide, methyl propenyl disulfide, methyl-2-propenyl disulfide, dipropyl disulfide, propyl propenyl disulfide, propyl propenyl disulfide, propyl-2-propenyl disulfide, dimethyl trisulfide, diisopropyl trisulfide, propyl propenyl trisulfide, propyl propenyl trisulfide, 2,5-dimethyl thiophene, 4 3,4- or 2,4-dimethyl thiophene | | dynamic headspace method | Gas Chromatography-Olfactometry (GC/MS-O) | | | [86] | |
| Hybrid between Onion (*Allium cepa* L.) and Leek (*Allium porrum* L.) | | | 1-propanethiol, dimethyl disulfide, 3,2-methyl 2-pentenal, methyl propyl disulfide, dimethylthiophene, (Z)-methyl 1-propenyl disulfide, (E)-methyl 1-propenyl disulfide, dipropyl disulfide, dimethyl trisulfide, (Z)-1propenyl propyl disulfide, (E)-1propenyl propyl disulfide, methyl propyl trisulfide, dipropyl trisulfide | | simultaneous distillation/extraction method | gas chromatography (GC) and GC/mass spectrometry | | | [87] | |

References

1. Yang, M.H.; Kim, N.H.; Heo, J.D.; Rho, J.R.; Ock, K.J.; Shin, E.C.; Jeong, E.J. Comparative Evaluation of Sulfur Compounds Contents and Antiobesity Properties of *Allium hookeri* Prepared by Different Drying Methods. *Evid. Based Complement. Altern. Med*. **2017**, *2017*, doi:10.1155/2017/2436927.
2. Radulović, N.S.; Miltojević, A.B.; Stojković, M.B.; Blagojević, P.D. New volatile sulfur-containing compounds from wild garlic (*Allium ursinum* L., *Liliaceae*). *Food Res. Int.* **2015**, *78*, 1–10.
3. Nguyen, T.; Sa, A.; Van, N.; Mot, M.; Thuc, N.T.; Nguyen, T.; Diem, T. GC/MS-TOF analysis of essential oil composition of the *Allium schoenoprasum* L. bulbs cultivated from quang tri-vietnam. *Tạp Chí Khoa Học Công Nghệ Và Thực Phẩm* **2019**, 18, 12–22.
4. Pino, J.A.; Fuentes, V.; Correa, M.T. Volatile constituents of Chinese chive (*Allium tuberosum* Rottl. ex Sprengel) and rakkyo (*Allium chinense* G. Don). *J. Agric. Food Chem.* **2001**, *49*, 1328–1330.
5. Satyal, P.; Craft, J.; Dosoky, N.; Setzer, W. The Chemical Compositions of the Volatile Oils of Garlic (*Allium sativum*) and Wild Garlic (*Allium vineale*). *Foods* **2017**, *6*, 63.
6. Hoseinpoor, M.E.M.F. Recognition of Sulfur Compounds in Tissue Culture Different Organs of Persian Shallot (*Allium hirtifolium* Boiss) by GC/MS. *J. Herb. Drugs* **2016**, *6*, 219–225.
7. Nielsen, G.S.; Poll, L. Determination of Odor Active Aroma Compounds in Freshly Cut Leek (*Allium ampeloprasum* Var. Bulga) and in Long-Term Stored Frozen Unblanched and Blanched Leek Slices by Gas Chromatography Olfactometry Analysis. *J. Agric. Food Chem*. **2004**, *52*, 1642–1646.
8. Schulz, H.; Krüger, H.; Liebmann, J.; Peterka, H. Distribution of Volatile Sulfur Compounds in an Interspecific Hybrid between Onion (*Allium cepa* L.) and Leek (*Allium porrum* L.). *J. Agric. Food Chem.* **1998**, *46*, 5220–5224.
