# Supplementary material for: Comparison of Organosulfur and Amino Acid Composition between Triploid Onion Allium cornutum Clementi ex Visiani, 1842, and Common Onion Allium cepa L., and Evidences for Antiproliferative Activity of Their Extracts
Source: Plants (Basel). 2020 Jan 13;9(1):98. doi: 10.3390/plants9010098 (PMC7020437; doi:10.3390/plants9010098)
Supplement: Supplementary file 1 [file plants-09-00098-s001.zip › plants-677263-supplementary-1/Data S7_Table 4.docx]

**Table 4.** Retention time (RT), limit of detection (LOD), limit of quantification (LOQ) and coefficient of determination (R^2^) for amino acids.

| **Amino acid** | **Retention time (RT) (min)** | **Limit of detection (LOD)**  **nmol/ml** | **Limit of quantification**  **(LOQ)**  **nmol/ml** | **R^2^** |
| --- | --- | --- | --- | --- |
| Asp (Aspartic acid) | 18,78 | 0,10 | 0,30 | 0,9940 |
| Ser (Serine) | 20,43 | 0,07 | 0,21 | 0,9952 |
| Glu (Glutamic acid) | 21,28 | 0,07 | 0,21 | 0,9956 |
| Gly (Glycine) | 22,32 | 0,06 | 0,18 | 0,9959 |
| His (Histidine) | 22,98 | 0,03 | 0,09 | 0,9962 |
| Arg (Arginine) | 25,14 | 0,03 | 0,09 | 0,9965 |
| Thr (Threonine) | 25,39 | 0,03 | 0,09 | 0,9968 |
| Ala (Alanine) | 26,30 | 0,03 | 0,09 | 0,9966 |
| Pro (Proline) | 27,90 | 0,07 | 0,21 | 0,9951 |
| Cys (Cysteine) | 30,89 | 0,08 | 0,24 | 0,9965 |
| Tyr (Tyrosine) | 31,13 | 0,03 | 0,09 | 0,9960 |
| Val (Valine) | 32,16 | 0,02 | 0,06 | 0,9951 |
| Met (Methionine) | 32,61 | 0,02 | 0,06 | 0,9940 |
| Lys (Lysine) | 34,89 | 0,04 | 0,12 | 0,9955 |
| Ileu (Isoleucine) | 35,43 | 0,01 | 0,03 | 0,9944 |
| Leu (Leucine) | 35,86 | 0,01 | 0,03 | 0,9927 |
| Phe (Phenylalanine) | 36,70 | 0,01 | 0,03 | 0,9992 |
